# An Immunological Approach to the Biocompatibility of Mesoporous SiO_2_-CaO Nanospheres

**DOI:** 10.3390/ijms21218291

**Published:** 2020-11-05

**Authors:** María Montes-Casado, Adrian Sanvicente, Laura Casarrubios, María José Feito, José M. Rojo, María Vallet-Regí, Daniel Arcos, Pilar Portolés, María Teresa Portolés

**Affiliations:** 1 Centro Nacional de Microbiología, Instituto de Salud Carlos III, Majadahonda, 28220 Madrid, Spain; mmontes@isciii.es; 2 Departamento de Bioquímica y Biología Molecular, Facultad de Ciencias Químicas, Universidad Complutense de Madrid. Instituto de Investigación Sanitaria del Hospital Clínico San Carlos (IdISSC), 28040 Madrid, Spain; adriansanvicenteg@gmail.com (A.S.); laura.casarrubios.molina@gmail.com (L.C.); mjfeito@ucm.es (M.J.F.); 3 Departamento de Medicina Celular y Molecular, Centro de Investigaciones Biológicas, CSIC, 28040 Madrid, Spain; jmrojo@cib.csic.es; 4 Departamento de Química en Ciencias Farmacéuticas, Facultad de Farmacia, Universidad Complutense de Madrid, Instituto de Investigación Sanitaria Hospital 12 de Octubre i+12, Plaza Ramón y Cajal s/n, 28040 Madrid, Spain; vallet@ucm.es (M.V.-R.); arcosd@ucm.es (D.A.); 5 CIBER de Bioingeniería, Biomateriales y Nanomedicina, CIBER-BBN, 28040 Madrid, Spain; 6 Presidencia, Consejo Superior de Investigaciones Científicas, 28006 Madrid, Spain

**Keywords:** mesoporous bioactive glass, nanomaterial, T cell, B cell, dendritic cell, dendritic cell maturation, phosphoinositide 3-kinase PI3-K, ICOS, endocytosis

## Abstract

Mesoporous bioactive glass nanospheres (NanoMBGs) have high potential for clinical applications. However, the impact of these nanoparticles on the immune system needs to be addressed. In this study, the biocompatibility of SiO_2_-CaO NanoMBGs was evaluated on different mouse immune cells, including spleen cells subsets, bone marrow-derived dendritic cells (BMDCs), or cell lines like SR.D10 Th2 CD4^+^ lymphocytes and DC2.4 dendritic cells. Flow cytometry and confocal microscopy show that the nanoparticles were rapidly and efficiently taken up in vitro by T and B lymphocytes or by specialized antigen-presenting cells (APCs) like dendritic cells (DCs). Nanoparticles were not cytotoxic and had no effect on cell viability or proliferation under T-cell (anti-CD3) or B cell (LPS) stimuli. Besides, NanoMBGs did not affect the balance of spleen cell subsets, or the production of intracellular or secreted pro- and anti-inflammatory cytokines (TNF-α, IFN-γ, IL-2, IL-6, IL-10) by activated T, B, and dendritic cells (DC), as determined by flow cytometry and ELISA. T cell activation surface markers (CD25, CD69 and Induced Costimulator, ICOS) were not altered by NanoMBGs. Maturation of BMDCs or DC2.4 cells in vitro was not altered by NanoMBGs, as shown by expression of Major Histocompatibility Complex (MHC) and costimulatory molecules (CD40, CD80, CD86), or IL-6 secretion. The effect of wortmannin and chlorpromazine indicate a role for phosphoinositide 3-kinase (PI3K), actin and clathrin-dependent pathways in NanoMBG internalization. We thus demonstrate that these NanoMBGs are both non-toxic and non-inflammagenic for murine lymphoid cells and myeloid DCs despite their efficient intake by the cells.

## 1. Introduction

Among the growing number of nanomaterials with therapeutic potential, mesoporous silica nanoparticles (MSN) have gained the attention of the nanomedicine research community, especially for the potential treatment of different biomedical challenges, including cancer, infectious processes and others [1,2,3,4,5,6].

Mesoporous silica nanoparticles have unique, advantageous physical-chemical properties that make them ideal platforms to design multifunctional nanosystems [7]. These include well-ordered internal mesopores with large pore volume and surface area, tunable size and shape, robustness and easy surface modification. The diameter of the pores determines the size of the biologically active molecules that can be loaded within the mesopores and the rate of release, controlling the diffusion of the molecules into the surrounding physiological medium. Silica-based mesoporous nanoparticles present low toxicity and high biocompatibility, easy functionalization and great loading capacity of many different types of therapeutic agents within their pores, favoring the possibility of modifying the surface to target the particles to the malignant areas. The advantage of these nanomaterial formulations over conventional systems is that they can increase treatment efficacy and decrease side effects through their precise targeting mode of action [7,8,9,10]. Another key challenge is to prevent the early release of the loaded agent before the target is reached, for which mesopores can be blocked by gates that respond to stimuli whose activation would allow the drug to be released. It is also possible to design multifunctional nanoparticles with synergistic effects for improved dual therapies [7].

Whatever the design of the MSN nanoparticles, the biocompatibility of these nanosystems must be evaluated before their application in the body. Biocompatibility of a material involves it being non-cytotoxic yet it induces the cellular response for which it has been designed. The ultimate functional success or failure of the biomaterials after their incorporation into the body depends invariably on the host tissue response and therefore they must not induce immune reaction which might reduce healing or produce rejection by the body [11,12]. Thus, the in vitro evaluation of the biomaterial effects on immune system is an essential aspect of biocompatibility assessment [13,14].

The immune response comprises both innate and adaptive defense mechanisms, with the coordinated participation of different cell populations. The innate immune system provides the first line of defense against foreign microbes and particulate materials; and involves the action of neutrophils, monocytes, dendritic cells and macrophages which carry out phagocytosis and produce reactive oxygen species, antimicrobial peptides and inflammatory mediators. Engineered nanoparticles can interact with the immune system in many different ways. The contact of body fluids and blood cells with biomaterials may trigger the activation of granulocytes, monocytes and macrophages, driving to an acute and lately chronic inflammatory response.

Once the nanomaterial has been released into a biological medium it becomes modified. When in contact with body fluids, nanoparticles can undergo significant surface changes, as they can adsorb different proteins and other plasma molecules depending of their hydrophobicity. Thus, a bio-corona coating the surface of nanoparticles is formed that determines the mode of interaction with innate cells, the recognition of the foreign material and its elimination or tolerance [15,16]. The effect of the contact of the biomaterial surface and the cell surface depends on different properties of the biomaterial. Nanoparticle characteristics such as size, shape and deformability may influence their uptake by immune cells and subsequent immune responses [15,16,17,18]. Thus, rough or smooth nanomaterials provoke different cells reactions. Furthermore, the presence of nanospikes on the biomaterial surface produces mechanical stress on the immune cells which tune the activation of the innate immune response [18]. Nanoparticles may interact with and become internalized by phagocytic cells as macrophages or dendritic cells, key antigen-presenting cells (APCs) of the immune system modulating the inflammasome activation and tuning the innate immune response. So, efforts are being made to engineer nanoparticles in order to modulate immunogenicity and adjuvanticity [15,18].

Dendritic cells (DCs) are specialized APCs of the innate immune system which locate in most peripheral and mucosal tissues where they constantly monitor their ambient surroundings, taking up cell debris and foreign materials. Their status as professional APCs serves to link the innate immune response to an antigen-specific adaptive response. These cells are armed with pattern-recognition receptors (PRR) that allow for the detection of invading pathogens or signs of cell stress and damage. Ligation of these receptors results in maturation of DCs associated with morphological changes, the expression of costimulatory molecules (namely, CD80, CD86, CD40), the release of proinflammatory cytokines and the increase of surface expression of molecules of the Major Histocompatibility Complex (MHC) that harbor antigenic peptides [19,20].

Mature DCs migrate to lymph nodes where they act as professional APCs that interact with and stimulate naive T lymphocytes providing them with the three types of signals required for T cells to be activated and become effector T cells [19,20,21]. These three types of signals come from: (i) antigen-specific T cell receptor interaction in the context of MHC plus peptide antigen complexes expressed on the APC, with the collaboration of CD4 or CD8 co-receptors on the T cell; (ii) costimulatory signaling mediated by B7 family members (CD80, CD86) on the DC acting as ligands of CD28 on the T cell; and (iii) cytokine-mediated signaling promoted by the secretion of these soluble mediators from the APC, which are determinant in the T cell differentiation. In this process, a cross-talk between APC and T cell takes place, provoking signals in both types of cells leading to the activation of the T lymphocytes, that enter a differentiation process to act as effector immune cells of different CD4^+^ T helper (Th) phenotypes (i.e., Th1, Th2, Th17, Tfh). Th cells produce inflammatory cytokines and collaborate with B lymphocytes to mount an adaptive immune response producing antigen-specific antibodies (Ab) [19,20,21]. APCs also interact with CD8^+^ cytotoxic T cells to promote their antigen-specific effector lytic function.

DCs efficiently detect and engulf various kinds of nanoparticles both in vitro and in vivo [11,13,15,16,17,22,23]. Depending on the way DCs are activated, T cells are subsequently stimulated to differentiate into distinct lineages of T helper cells, primarily including Th1, Th2 and Th17, which lead to cellular immunity, humoral immunity and tissue inflammation, respectively. Recent studies revealed that nanoparticles can affect all steps of DC induced immunity [17,23]. Largely, the interaction of biomaterials with host immune cells, including DC, macrophages and T or B lymphocytes, may have negative implications for tissue remodeling, highlighting the importance of immune system compatibility studies.

Given the great potential application of mesoporous SiO_2_-CaO nanospheres (NanoMBGs) in different fields, the present study is focused on the in vitro interaction of these nanospheres with immune cells involved in both innate and acquired immunity. The novelty of this work is related to the evaluation of the biocompatibility of these NanoMBGs through the analysis of their effects on the viability, activation and maturation processes of immune cells with key functions, including dendritic cells (which connect innate and adaptive immunity) and T and B lymphocytes (which constitute the effector arm of adaptive immunity). An exhaustive analysis of the specific markers and pro- or anti-inflammatory cytokines of each cell type has been carried out, as well as the molecular mechanisms of entry of these nanoparticles into these different cell types of the immune system.

## 2. Results and Discussion

### 2.1. Incorporation of NanoMBGs by Murine Spleen Cells

**Figure 1:**
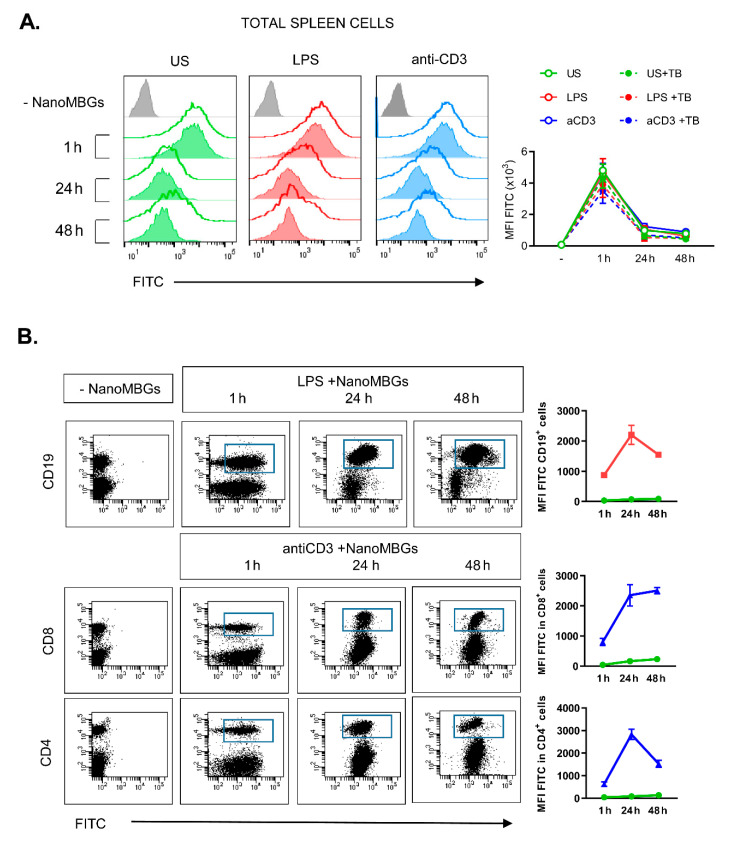
Incorporation of mesoporous bioactive glass nanospheres (NanoMBGs) by murine spleen cells. (**A**) Representative histograms showing the incorporation of NanoMBGs labeled with fluorescein isothiocyanate (FITC-NanoMBGs) in total murine spleen cells with different stimuli at the indicated times. Total spleen cells were stimulated with LPS (middle panel, red) or anti-CD3 Ab (right panel, blue) or left unstimulated (US, left panel, green) during the time indicated. Grey: Control cells without nanospheres. Solid lines: Cells incubated with NanoMBGs. Filled histograms: Cells with NanoMBGs and trypan blue, to confirm that NanoMBGs are inside the cell and not adsorbed on cell surface. (**B**) Representative dot-plots and graphs of one representative experiment out of three performed, showing the FITC-NanoMBGs incorporation in B (CD19) or T (CD4 and CD8) spleen cell subpopulations with different stimuli. (**A**,**B**) Right, analysis of median of fluorescence intensity (MFI) of cells treated as indicated in the legend. Data from one representative experiment out of three performed are shown.

The spleen is a lymphoid organ which contains different types of immune cells, including T- and B-lymphocytes (about 80% in the mouse spleen), macrophages and other APCs (about 10%) and natural killer (NK) cells [24]. These cells circulate through the body monitoring for signals which trigger an innate response or detecting non-self antigens, thus becoming activated, differentiated and expanded, mounting an acquired immune response. T cell activation via antigen presentation does not occur in contact with a biomaterial if the biomaterial is not degradable or if no bacteria transiently attach to its surface. However, it has been suggested that synthetic biomaterials present functional groups on their surfaces acting as mitogens which can polyclonally trigger lymphocytes by cell surface glycoprotein cross-linking [11,25]. We assessed this possibility studying the effects of NanoMBGs on normal murine spleen cell suspensions in vitro. First, we assessed if these nanospheres were incorporated by the main spleen cell subsets. With this objective, NanoMBGs labeled with fluorescein isothiocyanate (FITC-NanoMBGs) were cultured for different times with no additional stimulus, or activated with LPS (a bacterial endotoxin, stimulator of TLR4 expressed by macrophages, DCs and B lymphocytes among others), or with anti-CD3 antibody (a polyclonal stimulator of T lymphocytes). FITC-NanoMBGs were readily incorporated in the total spleen cells in the absence of added stimulus and in the presence of LPS or anti-CD3, as measured by the median of fluorescence intensity (MFI) (Figure 1A). Incubation of the FITC-NanoMBGs with the cells in the presence of trypan blue ruled out the possible FITC-biomaterial adsorption on the cell surface (Figure 1A), concluding that the FITC-NanoMBGs were really uptaken into the cells. Analysis of the different spleen cell populations (Figure 1B) indicated that both T (CD4^+^ or CD8^+^ subpopulations) and B (CD19^+^) lymphocytes incorporate the FITC-NanoMBGs when the corresponding stimulus is present, as well as in the absence of added stimuli (not shown).

### 2.2. Effects of NanoMBGs on Cell Subpopulations, Cytokine Expression and Proliferation of Activated Mouse Spleen Cells

Activation of normal resting lymphocytes promotes their differentiation to different effector subpopulations producing specific cytokines. To further study the potential effect of FITC-NanoMBGs on these processes, we analyzed the effect of nanospheres on the balance of lymphoid subpopulations (measured by specific surface markers) and cytokine secretion (measured as intracellular interleukin expression).

**Figure 2:**
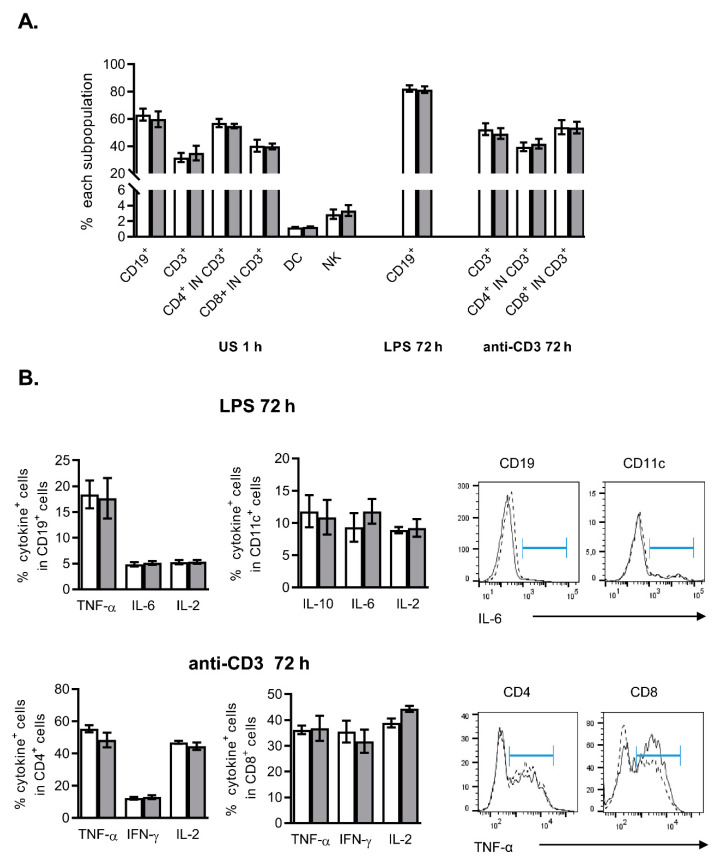
NanoMBGs do not alter the cell subpopulations balance or cytokine expression in stimulated mouse spleen cells. (**A**) Analysis of the different subpopulations in spleen cells unstimulated (US, 1 h) or with the indicated stimuli (72 h) in the presence (grey bars) or absence (white bars) of FITC-NanoMBGs. Unstimulated cells were incubated with FITC-NanoMBGs during 1 h. Mean ± SEM of one experiment out of two, each with three biological samples, is shown. (**B**) Analysis of cytokines produced in the spleen subpopulations shown in (**A**), in the presence (grey bars/solid line) or absence (white bars/dashed line) of NanoMBGs. Right, histograms showing the expression of IL-6 in CD19^+^ or CD11c^+^ cells (upper histograms) and TNF-α in CD4^+^ or CD8^+^ T cells (bottom histograms) from one representative experiment.

Figure 2A shows that the frequencies of different spleen subsets such as T cells (CD3^+^, CD3^+^CD4^+^, CD3^+^CD8^+^) or B cells (CD19^+^), natural killer (NK1.1^+^) or dendritic cells (CD11c^+^) were not significantly altered by the presence of FITC-NanoMBGs in the absence of stimulus, during 1 h. The nanomaterial did not alter the frequencies of the different spleen cells upon 72 h activation of either T cells (mediated by anti-CD3 Ab) or APCs (by LPS). Cytokine expression and secretion constitute an important effector mechanism of immune cells, determining subsequent activation or suppression of other cell subpopulations. Among them, we have analyzed IL-2, IL-6, IL-10, interferon-γ (IFN-γ) and tumor necrosis factor α (TNF-α) because of their important role in immune response and inflammation. To analyze potential secondary effects of NanoMBGs on immune cells, we studied the intracellular expression of these cytokines in spleen cells activated for 72 h with a B cell mitogen and APC activator (LPS) (Figure 2B upper panels) or a T cell stimulus (anti-CD3) (Figure 2B bottom panels). No significant effects of FITC-NanoMBGs on the intracellular expression of the growth/activating factor IL-2, the inflammatory mediators IL-6, IFN-γ or TNF-α, or the anti-inflammatory/regulatory factor IL-10 were detected in these conditions.

**Figure 3:**
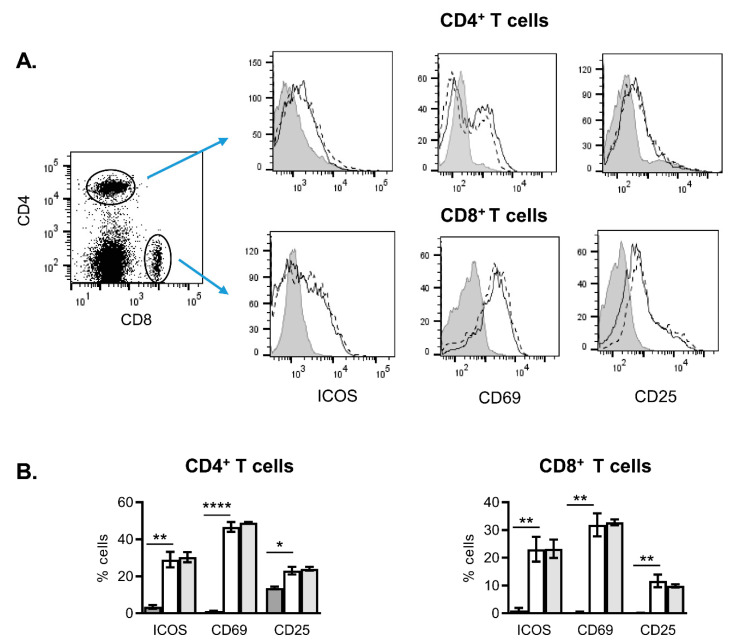
Effect of NanoMGBs on primary T cell activation. Analysis of activation markers (Inducible Costimulator, ICOS, CD25 and CD69) in spleen T-cells, activated for 24 h with anti-CD3 Ab. (**A**) Representative histograms showing the surface expression of ICOS, CD69 and CD25, in unstimulated (grey) spleen CD4^+^ or CD8^+^ T cells or after 24 h of anti-CD3 stimulus; in the absence (dashed) or presence (solid) of FITC-NanoMBGs. (**B**) Graphs showing percentage of cells positive for ICOS, CD69 or CD25 markers in CD4^+^ or CD8^+^ T cells. Basal expression (dark grey bars) or expression in activated cells in the absence (white bars) or presence (light grey bars) of FITC-NanoMBGs are shown. Mean ± SEM of one experiment out of three, each with three biological samples, is shown. Asterisks indicate significant difference when activated vs. non-activated cells are compared. * *p* < 0.05; ** *p* < 0.01; **** *p* < 0.0001.

T cell stimulation induces the expression of various activation markers in the cell surface within 24 h, including CD25 (α chain of IL-2 receptor), CD69 (an early activation marker) and Inducible Costimulator (ICOS) which mediate cell growth/activation and modulate the acquisition of effectors’ functions through specific intracellular signalling. We have further studied the effect of NanoMBGs on the expression of these markers by activated spleen T cells (Figure 3). Spleen cells were stimulated for 24 h with anti-CD3 Ab in the presence or absence of FITC-NanoMBGs. As expected, the expression of ICOS, CD69 and CD25 was increased in the surface of activated CD4^+^ and CD8^+^ T lymphocytes. No significant effect of NanoMBGs was observed concerning the upregulation of these activation markers.

**Figure 4:**
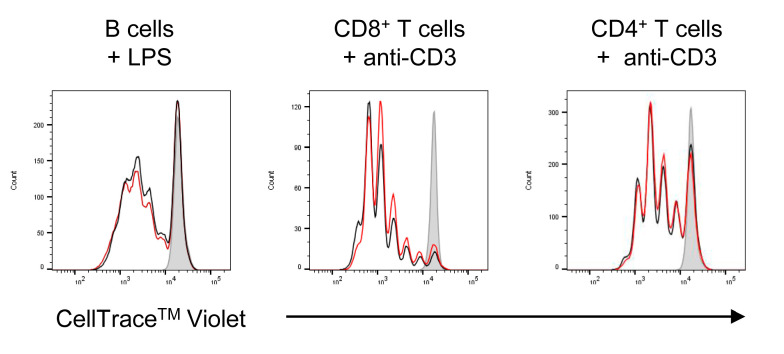
Effects of NanoMBGs on the proliferation of different subpopulations of spleen cells. Spleen cells were cultured for 72 h in the presence of different stimuli (LPS or anti-CD3 Ab); CellTrace^TM^ Violet was used to assess the proliferation capacity of each subpopulation. The effect of different stimuli in the presence (red line) or absence (black line) of FITC-NanoMBGs was analyzed. Grey: unstimulated control. Results of one representative experiment out of three performed are shown here.

We have not observed any significant effect of NanoMBGs at early stages of the immune cell activation; however, these NanoMBGs are truly incorporated into the cells and we decided to further analyze if they could interfere in cell division. Thus, the effects of NanoMBGs on the proliferation of different spleen cell subpopulations were studied. Figure 4 shows that no effect of this nanomaterial was found in LPS-induced B lymphocyte proliferation or in anti-CD3-induced proliferation of CD4^+^ or CD8^+^ lymphocytes after 72 h of incubation. CD19, CD4 and CD8 surface labeling was used to specifically detect B, CD4^+^ or CD8^+^ T cells, respectively.

### 2.3. Effects of NanoMBGs on the Th2 CD4^+^ SR.D10 Murine Cell Line

**Figure 5:**
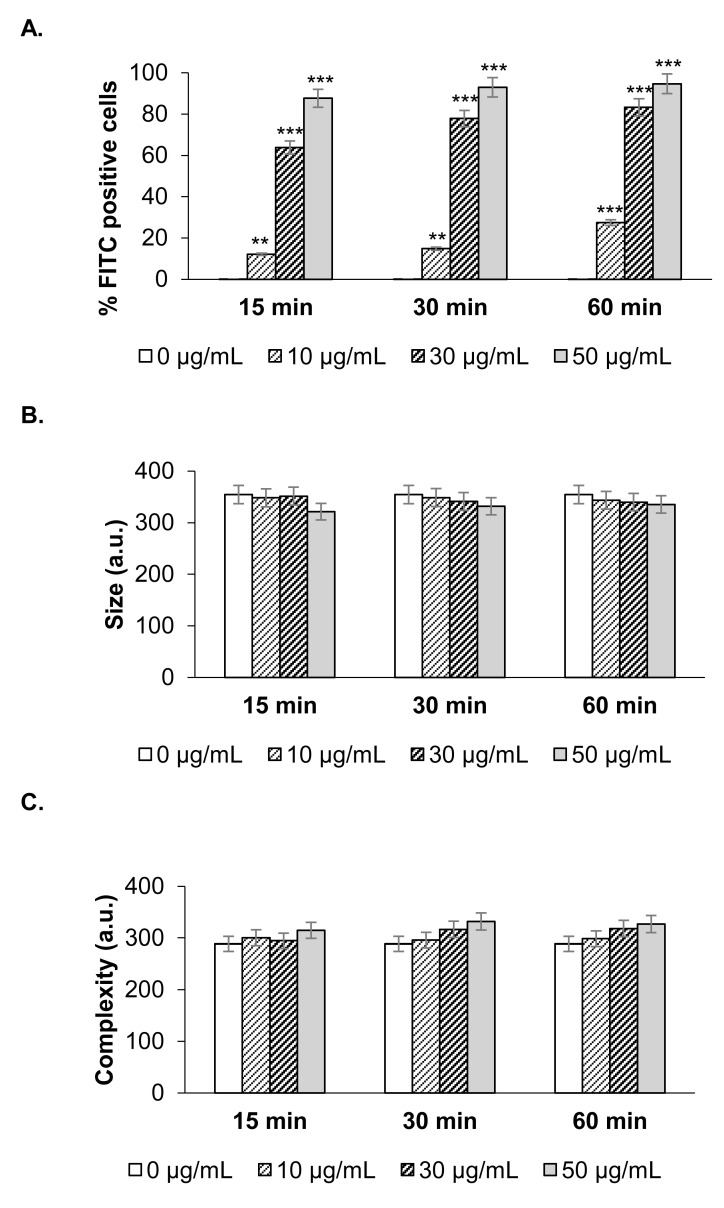
Incorporation of FITC-NanoMBGs by Th2 CD4^+^ SR.D10 cell line. Analysis of nanomaterial incorporation in SR.D10 cells which were incubated with a range of FITC-NanoMBG concentrations, during the time indicated in the figure. (**A**) Frequency of FITC positive cells. (**B**) Analysis of size and (**C**) complexity of the cellular population. Data (mean ± SEM) from one representative experiment out of three, each with triplicates, are shown. Asterisks indicate significant difference when cells in the presence or absence of NanoMBGs are compared. ** *p* < 0.01; *** *p* < 0.001.

We have shown that these NanoMBGs are readily incorporated into murine primary spleen cells with no significant changes in their activation and proliferation. We have also studied the possible incorporation of this nanomaterial into the differentiated Th2 CD4^+^ T cell line SR.D10. The time course of different doses of NanoMBGs incorporation was analyzed, showing a fast, dose-dependent uptake of the nanomaterial, as early as 15 min of incubation (Figure 5A). No changes in cell size and complexity were found whatever the FITC-NanoMBGs dose used in the assay was (Figure 5B,C).

### 2.4. Effects of NanoMBGs in Murine Bone Marrow-Derived Dendritic Cells during the Maturation Process

**Figure 6:**
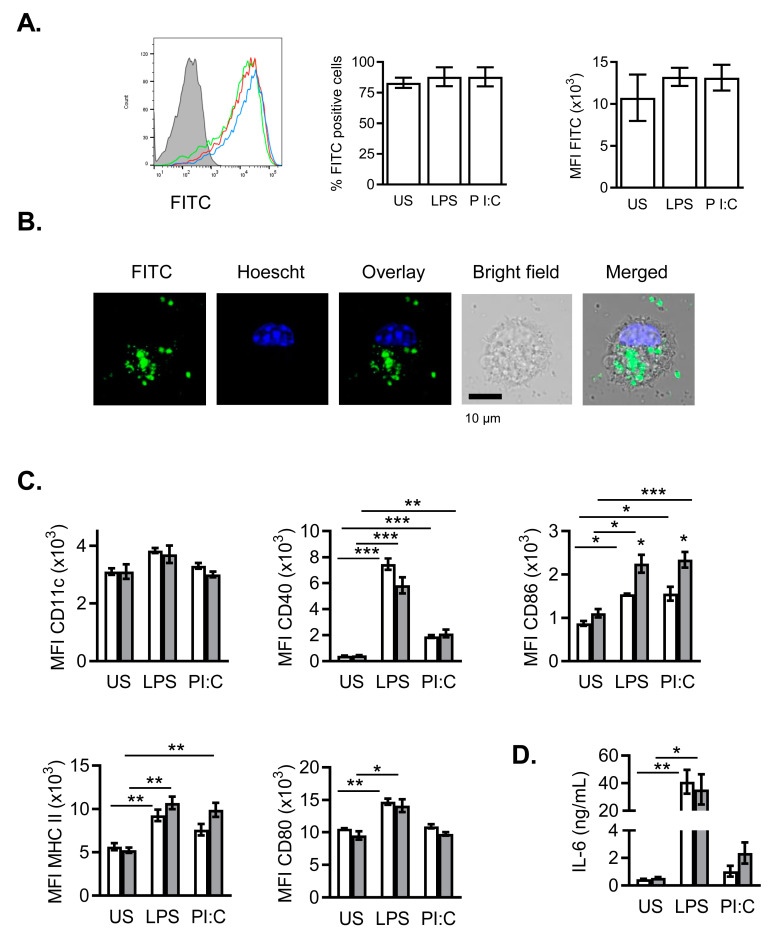
Incorporation of FITC-NanoMBGs by bone marrow-derived dendritic cells (BMDCs). (**A**) Incorporation of nanospheres (histogram and graphs) into BMDCs with different stimuli during 24 h. Cytometry analysis (percentage of positive cells and median of fluorescence intensity, MFI) of BMDCs treated with FITC-NanoMBGs plus different stimuli such as LPS (red) or Poly I:C (PI:C, blue) or unstimulated cells (green, US) are shown. BMDCs in the absence of nanospheres are depicted in grey. (**B**) Confocal microscopy analysis of FITC-NanoMBGs incorporation into BMDCs after 2 h of incubation. Images of a representative 0.772 µm slice are shown. Panels (from left to right) show FITC-nanomaterial (green), cell nucleus stained with Hoescht (blue) and the overlay of both. Bright field image of the cell and the merged images are also shown. (**C**) BMDCs were activated as in (A) in the presence (grey bars) or absence (white bars) of NanoMBGs, and the expression of different surface markers (CD11c, CD40, CD80, CD86 and Class-II Major Histocompatibility Complex molecules (MHC II)) was analyzed by flow cytometry (median fluorescence intensity, MFI). (**D**) IL-6 production measured by ELISA in 24 h culture supernatants of BMDCs with (grey bars) or without (white bars) the NanoMBGs and the indicated stimuli. Data (mean ± SEM) from one representative experiment out of three, each with triplicates, are shown. Asterisks indicate significant difference when activated vs. non-activated cells are compared. * *p* < 0.05; ** *p* < 0.01; *** *p* < 0.001.

Dendritic cells are a key stone connecting innate and adaptive immune systems and are found in most tissues. The potency of DCs as professional antigen-presenting cells and regulators of the immune system reveals the importance of assessing their possible interaction with nanomaterials [17,23]. Thus, we have used bone marrow-derived dendritic cells, as a DC-enriched culture. We assessed the incorporation of FITC-NanoMBGs to bone marrow-derived dendritic cells (BMDCs) by FACS. Figure 6A shows that these nanospheres are incorporated into immature as well as into LPS- or Poly I:C-stimulated mature BMDCs (Figure 6A left). The corresponding cytometry analysis (Figure 6A, middle and right panels) is also shown. The maturation of the cells does not seem to affect their ability to incorporate the nanobeads. Confocal microscopy images taken in vivo of BMDCs incubated for at least 2 h with FITC-NanoMBGs corroborate the nanomaterial incorporation and its cytoplasmic distribution (Figure 6B).

Physicochemical properties of the nanoparticles, as size, shape and topography of the surface, are determinant for the recognition and uptake process by DCs and other immune cells [15,16,17,18,26]. NanoMBGs used in this study consist of monodisperse spherical nanoparticles of around 200 nm in diameter, although some nanoparticles have a certain degree of polyhedral morphology [8]. We show here that this nanomaterial is readily incorporated into immune cells of different nature, as T or B lymphocytes (Figure 1 and Figure 5) or bone marrow-derived dendritic cells of myeloid lineage (Figure 6). Stimulation of BMDCs through TLR-4 with LPS or TLR-3 with Poly I:C (Polyinosinic-polycitidylic acid, a mismatched double-stranded RNA), induces DC maturation concomitant with increased expression of MHC and costimulatory molecules on the cell surface, to facilitate DC-T cell interaction, signaling and cell activation. Figure 6C shows the increased surface expression of CD40, CD80, CD86 and MHC-II induced by LPS or Poly I:C in mature BMDCs. The presence of NanoMBGs did not significantly alter the expression of those surface molecules, except in the case of CD86 whose expression was slightly upregulated in the presence of the nanomaterial (Figure 6C).

Secretion of the pro-inflammatory IL-6 cytokine was strongly upregulated in BMDCs by LPS- or Poly I:C-mediated maturation, but no effect of the NanoMBGs was found (Figure 6D). It has been hypothesized that DCs use analogous mechanisms to recognize and respond to biomaterials as they are used to recognize and respond to pathogens [11]. Hence, DCs could initiate an immune response to biomaterials by recognizing “biomaterial associated molecular patterns”, analogous to “pathogen associated molecular patterns” (PAMPS), by using pattern recognition receptors (PRR), as TLRs or others damage associated molecular patterns (DAMPs). Ligation of these receptors results in the maturation of DCs associated with the upregulation of costimulatory molecules, MHC for Ag presentation and the release of proinflammatory cytokines including IL-6 among others [19,20]. In our study, neither IL-6 (Figure 6D) nor costimulatory ligands like CD80 and CD40 (Figure 6C) were induced or upregulated in immature BMDC (US) by NanoMBGs, showing that these nanospheres do not induce BMDC maturation by themselves.

Furthermore, our study of interaction of NanoMBGs with DCs allows us to check the absence of contaminant endotoxin or microbial bioproducts in the mesoporous SiO2-CaO nanospheres tested. The detection of endotoxins as contaminants in nanomaterials and pharmaceutical products is of particular importance, as it could lead to an immune reaction and septic shock [16] and different methods for endotoxin detection have been described and commercialized. Some of them are based on the use of DC lines as endotoxin sensors.

In the mature BMDCs, NanoMBGs did not modify the surface markers studied, nor enhance IL-6 secretion, and only a slight increase in CD86 expression was observed (Figure 6C,D). The CD86 increase might modulate subsequent DC-T cell interaction and activation. Although polarization of autologous T cells by biomaterial-treated DCs has been described in other systems [23], in this case further experiments could be necessary to assign its biological significance in vivo.

### 2.5. Effects of NanoMBGs on DC2.4 Murine Line

We further studied the effect of the NanoMBGs on DC2.4, a murine dendritic cell line that exhibits characteristic features of DCs including cell morphology, the expression of DC-specific markers and the ability to phagocytose and present exogenous antigens on both MHC class I and class II molecules [27].

**Figure 7:**
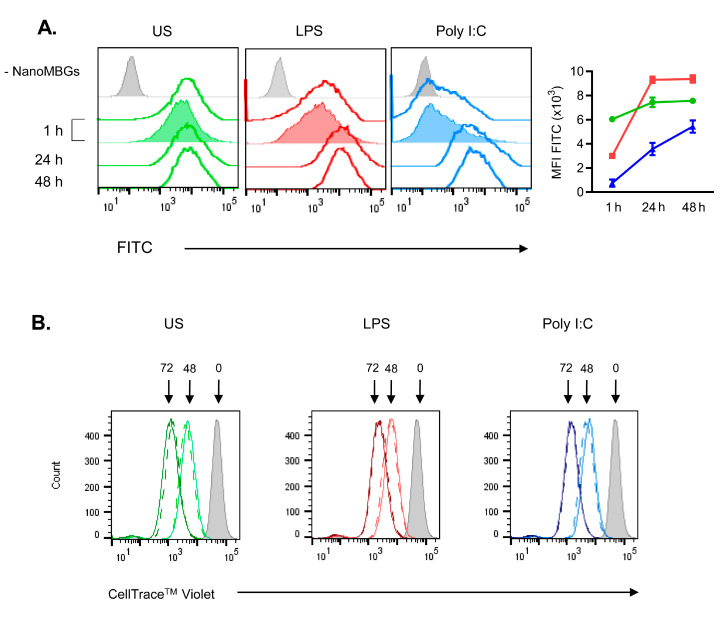
Incorporation of NanoMBGs into DC2.4 cells and effect on proliferation. (**A**) Time-course of the incorporation of FITC-NanoMBGs in DC2.4 cells cultured in the presence of different stimuli which promote dendritic cell maturation. Overlay histograms are representative of three experiments. DC2.4 cells were unstimulated (US, green) or LPS- (red) or Poly I:C (blue)-stimulated during 1, 24 or 48 h. In 1 h experiments, cells incubated with nanospheres and untreated (solid lines) or trypan blue-treated (green, red or blue filled histograms) are shown. Overlays show 24 h and 48 h results. Graph shows cytometry analysis (median of fluorescence intensity, MFI). (**B**) Overlay histograms showing DC2.4 cells proliferation measured by CellTrace^TM^ Violet during 48 and 72 h with LPS or Poly I:C or without (US) stimuli and in the presence (solid line) or absence (dashed line) of NanoMBGs. Data from one representative experiment out of three, each with triplicates, are shown.

A time course of intracellular FITC-NanoMBG uptake in DC2.4 cells is shown in Figure 7A. Unstimulated DC2.4 cells show a fast incorporation of the FITC-nanobeads (Figure 7A, US). At 1 h of incubation, a plateau of FITC-NanoMBG incorporation was reached until 48 h. At 1 h, a slight FITC-MFI decrease in the presence of trypan blue indicates that some nanobeads may still be attached to the outer cell surface (Figure 7A, left panel). No effect of trypan blue addition was found at 24 h or 48 h (results not shown). When DC2.4 cells were treated with LPS- or Poly I:C, TLR-induced maturation was obtained and a gradual, slower incorporation of the nanomaterial was detected, with a maximum at 24 h or later (Figure 7A, histograms and MFI FITC). These results suggest a concomitant decrease of the phagocytic capacity (especially in the presence of Poly I:C), a characteristic of DC maturation. In stimulated DC2.4, no alteration of FITC-MFI was found in the presence of added trypan blue at 1 h (Figure 7A, middle and right panels) or longer incubation (not shown), indicating that the NanoMBGs were effectively inside of the DC2.4 cells. When we analyzed the proliferative capacity of the DC2.4 cells in the presence of NanoMBGs (Figure 7B), we found no alterations at the different times or with the different maturation stimuli.

**Figure 8:**
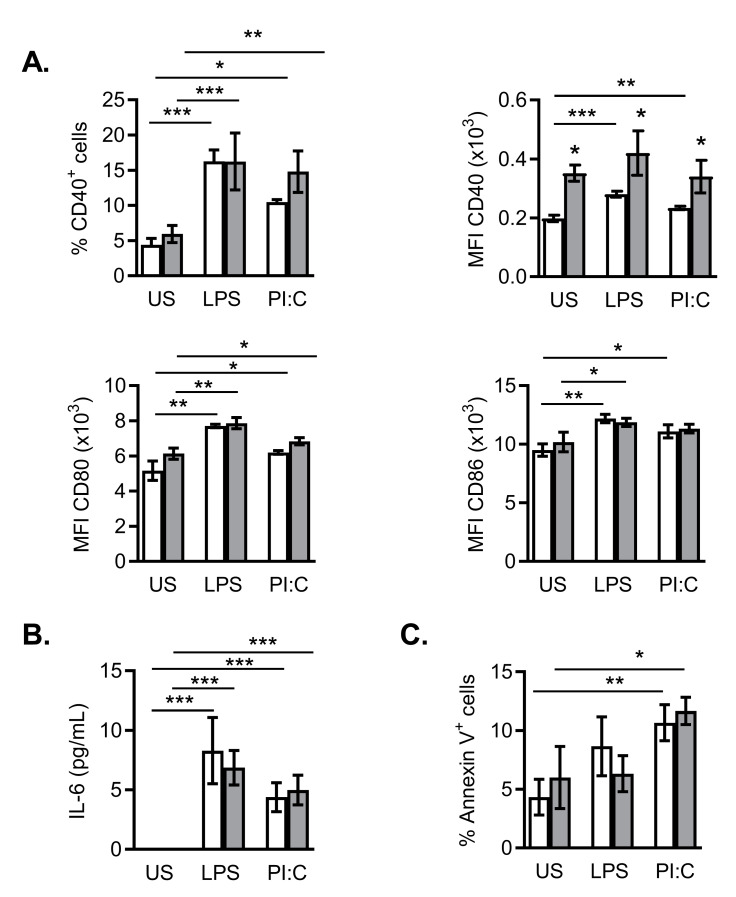
Effect of NanoMBGs in markers expression, IL-6 production and apoptosis in DC2.4 cells. (**A**) Expression (percentage of positive cells and median of fluorescence intensity, MFI) of different surface markers (CD40, CD80 or CD86) in DC2.4 cells, after 24 h of culture in the presence (grey bars) or absence (white bars) of NanoMBGs and different stimuli (LPS or Poly I:C). (**B**) IL-6 production measured by ELISA in culture supernatants of DC2.4 cells treated as in (**A**). (**C**) Spontaneous apoptosis in DC2.4 cells treated as in (**A**), measured by Annexin V assay. Data (mean ± SEM) from one representative experiment out of three, each with triplicates, are shown. Asterisks indicate significant difference between the indicated bars (activated vs. non-activated cells compared) or adjacent bars (plus vs. minus nanomaterial added to the cultures). * *p* < 0.05; ** *p* < 0.01; *** *p* < 0.001.

As mentioned before, maturation of DCs induced by the encounter with microbial derived bioproducts produces important changes in DC’s functional characteristics, namely, increase in costimulatory-ligands expression and secretion of cytokines. To further study the effect of the nanomaterial in the phenotype of the dendritic cell line DC2.4, we analyzed the surface expression of costimulatory-ligand markers as CD40, CD80 or CD86. Surface expression of these markers was increased in the presence of LPS or Poly I:C (Figure 8A). Whereas NanoMBGs did not change CD80 or CD86 expression levels, the nanoparticles increased CD40 fluorescence intensity in the presence or absence of LPS or Poly I:C in this cell line (Figure 8A).

Cytokine secretion is an important functional characteristic of mature DCs. Thus, maturation of DC2.4 cells was induced by addition of LPS or Poly I:C to the cultures and in the presence or not of the nanomaterial. Figure 8B shows enhanced IL-6 secretion when DC2.4 cells were maturated by either stimulus, but no effect of the nanomaterial. NanoMBGs alone did not induce IL-6 secretion in unstimulated DC2.4, also indicating the absence of contaminant endotoxin or microbial products in the studied nanomaterial.

The interaction of nanomaterials with cells may produce cell damage or toxicity leading to programmed cell death. To assess any possible effect of the nanomaterial on the spontaneous apoptosis of DC2.4 cell line, Annexin V assays were performed in stimulated cell cultures with or without added NanoMBGs. After 24 h of culture, no significant effect of the nanospheres on the spontaneous apoptosis of DC2.4 cell line was observed in any condition assayed (Figure 8C).

### 2.6. Mechanisms of NanoMBG Incorporation into the Cells

Knowledge of the interactions between nanoparticles and cell membranes is of great importance for the design of safe and efficient nanomedicines. In this study, we have shown that this nanomaterial not only interacts with the cell surface, but it is also taken by the cells. To analyze the incorporation of NanoMBGs into the DC2.4 cell line at the molecular level, we used different inhibitors that can interfere with the uptake processes of solid particles into the cells. Molecular targets of the inhibitors or cellular processes involved are shown in Table 1.

**Figure 9:**
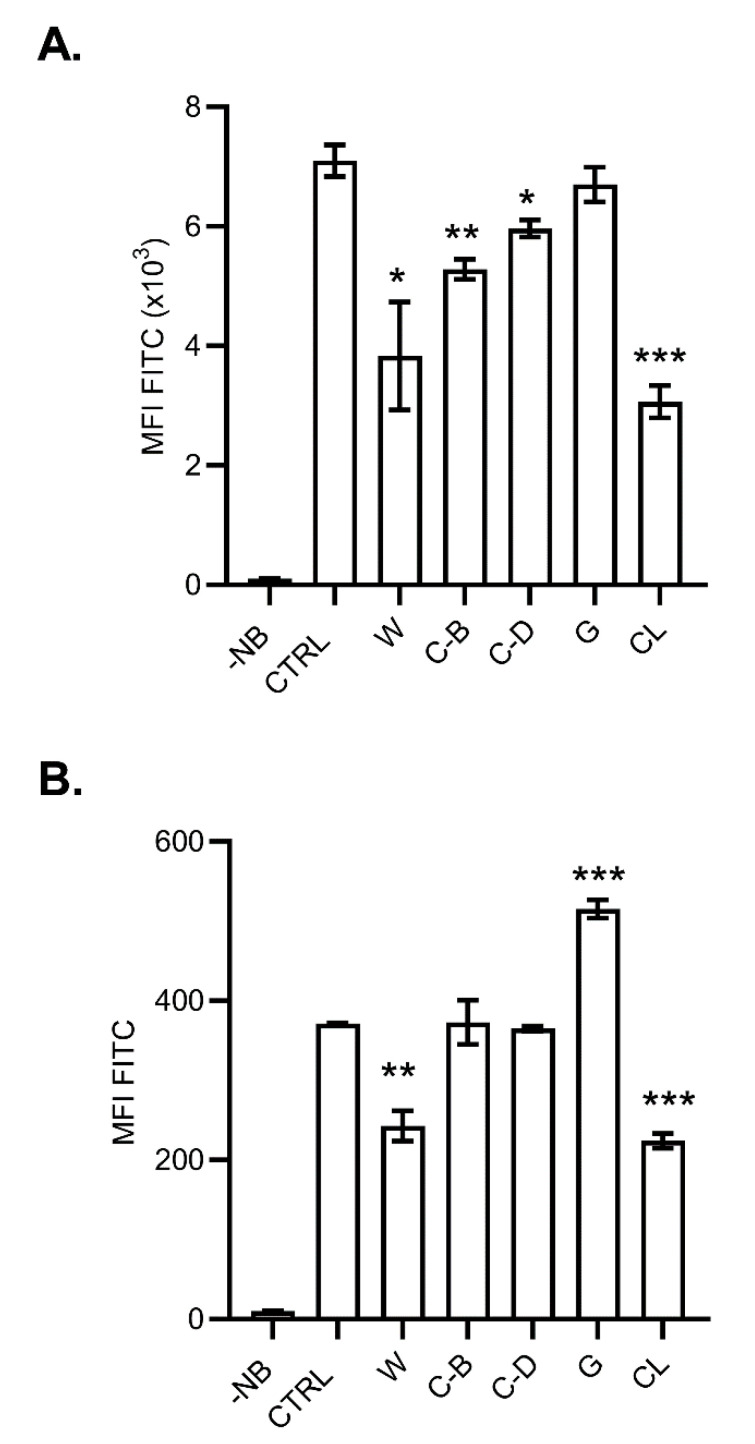
Effects of inhibitors in the FITC-NanoMBG incorporation by cells. Graphs showing the median of fluorescence intensity (MFI) in DC2.4 dendritic cell line (**A**) or Th2 CD4^+^ SR.D10 cell line (**B**) cultured for 2 h in the presence of NanoMBGs plus inhibitors as wortmannin (W), cytochalasin B (C-B), cytochalasin D (C-D), genistein (G) or chlorpromazine (CL) as described in the Methods section. CTRL, control without inhibitor; -NB, cells alone. Data from one representative experiment out of three, each with triplicates are shown. Asterisks (* *p* < 0.05; ** *p* < 0.01; *** *p* < 0.001) indicate significant differences as compared to CTRL without inhibitor.

Inhibitors were added to DC2.4 cells 2 h before the incubation with the NanoMBGs, and the cells were further cultured during 2 h before flow cytometry analysis (Figure 9A). Wortmannin (a pan-inhibitor of PI3Ks), cytochalasin B and D (actin filaments inhibitors) and chlorpromazine (which blocks clathrin-mediated endocytosis) inhibited the incorporation of FITC-NanoMBGs into DC2.4 cells. This would indicate that uptake of NanoMBGs in DC2.4 cell line is mediated by endocytosis mechanisms, such as phagocytosis or micropinocytosis, in which actin cytoskeleton, PI3K activity and clathrin are implicated. PI3K are a family of kinases which can mediate intracellular vesicular trafficking and through these actions contribute to a number of important physiological functions [33]. Different types of surface receptors expressed on DCs could interact with the NanoMBGs and provoke a clathrin-mediated endocytosis [26,34]. Genistein did not significantly modify FITC-nanobead internalization, indicating that caveolae-mediated endocytosis was not involved in NanoMBGs internalization in this cell line.

When the mechanisms of NanoMBG incorporation into SR.D10 Th2 CD4^+^ lymphocytes were analyzed (Figure 9B) we found an inhibitory effect of wortmannin and chlorpromazine, suggesting the participation of PI3 kinases and a clathrin-mediated endocytosis as in DC2.4 cells. However, no significant effects of cytochalasin B or D were found and an increased incorporation of nanomaterial was produced into SR.D10 cells in the presence of genistein. The action of this inhibitor seems to increase the entry of NanoMBGs into SR.D10 lymphocytes through other mechanisms that are not blocked for this treatment. Differences in NanoMBG internalization mechanisms between the DC2.4 dendritic cell line and the SR.D10 Th2 CD4^+^ cell line may be rooted in lineage differences (myeloid or lymphoid, respectively), surface receptor expression or signal mediator sensitivity to the different inhibitors. Genistein inhibits multiple tyrosine kinases, affecting various processes including endocytosis or chemotactic signalling. Genistein inhibits human memory T cell migration, but it can also directly trigger actin polymerization in memory CD4^+^ T cells at the same dose used in our study [31]. An altered actin polymerization induced by genistein might favor nanomaterial internalization in CD4^+^ T cell line SR.D10 (Figure 9B). In this regard, increased internalization of graphene oxide nanoparticles was reported in SAOS-2 cells after treatment with genistein [35]. The different inhibitory effects reported for genistein [30] may result in different outcomes depending on the system of study.

## 3. Materials and Methods

### 3.1. Synthesis and Characterization of FITC-NanoMBGs

NanoMBGs were prepared using a dual soft template strategy to obtain SiO_2_-CaO nanospheres comprising a large hollow core and radially distributed channels in the shell [35]. For this aim, 80 mg of PS-b-PAA were dissolved in 18 mL of tetrahydrofuran (THF) at room temperature under magnetic stirring (solution 1). Furthermore, 160 mg of hexadecyltrimethylammonium bromide (CTAB) were dissolved in 7.4 mL of D.I. water and 2.4 mL of ammonia (28% *w/v*) and stirred at 37 °C at 100 rpm in an incubator to avoid foaming of the solution (solution 2). After complete dissolution of both reactants, solution 1 was poured into solution 2 under vigorous stirring for 20 min. Thereafter the inorganic sources were added dropwise and step by step (25 μL of triethyl phosphate, (TEP), in 1.6 mL ethanol, 125 mg of Ca(NO_3_)_2_·4H_2_O in 1.6 mL of water and 0.52 of tetraethyl orthosilicate (TEOS) in 1.6 mL ethanol) at 20 min intervals. The mixture was covered with parafilm to avoid THF evaporation and stirred for 24 h at room temperature. Finally, the product was collected by centrifugation at 10,000× *g* rpm (g = 16.466) for 10 min and washed several times with an ethanol:water (1:1) solution. The resulting powder was dried and subsequently calcined at 550 °C for 4 h to remove the organic templates

For fluorescein isothiocyanate (FITC) labeling, aminopropyl triethoxysiliane (APTES) was dissolved in ethanol. Subsequently, 0.6 mg of fluorescein isothiocyanate were added and stirred for 5 h. This solution was added dropwise into the NanoMBG particles suspension, and the labeled particles were washed and collected by centrifugation.

Scanning electron microscopy (SEM) and transmission electron microscopy (TEM) images were collected with a JEOL F-6335 microscope (Jeol Ltd., Tokyo, Japan) and a JEOL-1400 microscope (Jeol Ltd., Tokyo, Japan), respectively. Chemical composition was determined by energy dispersive X-ray (EDX) spectroscopy during TEM observations. Surface area and porosity were determined by nitrogen adsorption analysis with a 3Flex (Micromeritics) analyzer. SEM micrographs and TEM images evidenced that NanoMBGs are small spheres of 200 nm in diameter, which contain a large hollow cavity at the core and a shell comprising mesopores radially distributed. The chemical composition determined by EDX spectroscopy was SiO_2_ 81.44-CaO 18.6 (% mol) and the nanospheres showed very high textural parameters with a surface area of 508.7 m^2^·g^−1^ and pore volume of 0.435 cm^3^·g^−1^ (see Supplementary Material).

For in vitro cultures, NanoMBGs were sterilized by immersion in absolute ethanol and desiccation before use.

### 3.2. Animals

Mice from C57BL/6 strain were bred under specific pathogen-free conditions in the animal care facility of the Instituto de Salud Carlos III (Majadahonda, Madrid, Spain), from stock purchased from Charles River. Sex-matched, 8–12 week old mice were used throughout the experiments. All experimental procedures were approved by the Ethics and Animal Welfare Committees of the Instituto de Salud Carlos III (OEBA-Majadahonda and CEIYBA). The study was carried out under project license PROEX 330/15 approved by the Consejeria de Medio Ambiente y Ordenación del Territorio de la Comunidad de Madrid. All the studies involving animals have been conducted according to local, national and European Union guidelines.

### 3.3. Culture and Activation of Murine Cells

Single cell suspensions from murine spleens were obtained as follows: After euthanasia of mice by CO_2_ inhalation, spleens were obtained, mechanically disrupted and the tissue was subsequently filtered through a 70 μm cell strainer (Corning). Erythrocytes were lysed with ammonium chloride buffer (0.75% NH_4_Cl, 20 mM HEPES, pH 7.2) and cell yield and viability were assessed by trypan blue exclusion in a Neubauer counting chamber. Spleen cells were then cultured in Click’s culture medium (CC) [36] supplemented with 10% heat-inactivated fetal calf serum (FCSi) at 37 °C in 5% CO_2._

To obtain murine bone marrow-derived dendritic cells, the method described by Inaba et al. [37] was followed with minor modifications. Briefly, after obtaining a sterile cell suspension from the bone marrow of mouse femurs, the cells were suspended in CC medium supplemented with granulocyte-macrophage colony-stimulating factor (GM-CSF) (20 ng/mL) and seeded on P-6 culture plates [38]. After 48 h of culture at 37 °C, 5% CO_2_, the plates were gently swirled (to remove nonadherent granulocytes) and 75% of the supernatant medium was aspirated trying not to drag the dendritic cells that grow forming colonies and remain loosely attached. The culture medium was replaced by the same volume of medium supplemented with GM-CSF. Two days later, medium supplemented with GM-CSF was added. After 5–8 days, the resulting loosely anchored colonies of bone marrow-derived dendritic cells were collected and used for the different experiments. BMDCs in this state were considered “immature” dendritic cells.

DC2.4 dendritic cell line [27] was generously given by M. Ramos. It was cultured in CC medium and treated with trypsin-EDTA 0.02% in sterile PBS to remove them from the culture flask. DC2.4 exhibits characteristic features of dendritic cells including cell morphology, the expression of dendritic cell-specific markers and the ability to phagocytose and present exogenous antigens on both MHC class I and class II molecules.

SR.D10 [39] is a clone obtained from the murine CD4^+^ Th2 cell line D10.G4.1 [40] specific for conalbumin fragment 134–146 bound to I–A^k^ class II major histocompatibility complex molecules. It was maintained as previously described [41], in Click’s medium supplemented with 10% FCSi containing 5 U/mL mouse recombinant (mr) IL-2, 10 U/mL mrIL-4 and 25 pg/mL mrIL-1. After 4 days, cultured cells were collected, exhaustively washed to eliminate added IL and used for in vitro assays.

In vitro cell activation was performed by adding the following stimuli to the cell cultures: Affinity purified Y-CD3-1 (anti-CD3 monoclonal Ab, 5 µg/mL, [42]); lipopolysaccharide (LPS) from *Escherichia coli* serotype R515, TLR grade (Enzo Biochem, Inc. Farmingdale, NY, USA) at 25 µg/mL for spleen cells assays or 100 ng/mL for DCs; or Poly I:C (1 µg/mL) (InvivoGen, Toulouse, France)..

For exposure to nanoparticles, the different cells were seeded in complete medium at a density of 1.0 × 10^6^/mL in 24- or 96-well tissue culture plates. Silica nanoparticles were added in CC medium at a final concentration of 50 µg/mL. After the indicated times, cells were analyzed.

### 3.4. Flow Cytometry Assays

Cell suspensions were stained in PBS with 5% FCSi, 0.05% sodium azide (staining buffer) and exhaustively washed before fluorescence-activated cell sorting (FACS). Fluorochrome-coupled antibodies against the surface antigens, CD3, CD4, CD8, CD11c, CD19, CD25, CD40, CD69, CD80, CD86, MHC-II, ICOS, NK1.1 and appropriate isotype controls were purchased from eBioscience (San Diego, CA, USA) or BioLegend (San Diego, CA, USA). Data were acquired on a FACSCalibur Becton Dickinson flow cytometer, or FACSCanto or BD LSRFortessa (BD Biosciences, San Jose, CA, USA) flow cytometers, and analyzed using FACSDiva (BD Biosciences) or FlowJo software (Tree Star, Ashland, OR, USA), version 10.0.

### 3.5. Analysis of Intracellular Cytokine Expression in Murine Spleen Cells

To analyze the effect of nanospheres on cytokine expression, suspensions of murine spleen cells were prepared as above. Furthermore, 2 × 10^6^ cells were seeded in 24-well culture plates and cultured for 72 h in the presence of sterilized FITC-nanospheres (50 µg/mL) and in the presence or not of different stimuli: Y-CD3-1 (anti-CD3 Ab, 5 µg/mL) or LPS (25 µg/mL), in 1 mL final volume. Then, the cells were treated as previously described [43]. Briefly, the cells were stimulated with PMA (20 ng/mL, (Sigma-Aldrich; St Louis, MO, USA) plus ionomycin (200 ng/mL, Sigma-Aldrich) and brefeldin A (10 μg/mL, Sigma-Aldrich) for 4 h and finally collected and processed for surface and intracellular cytokine detection by FACS. After staining of cell surface markers, the cells were fixed and permeabilized with a Cytofix/Cytoperm kit ((BD Biosciences) following the manufacturer´s instructions. Then, intracellular cytokines were stained using the following antibodies, all from eBioscience: PE-coupled anti-IL-2 (JES6-5H4), anti-IL-6 (MP5-20F3) and anti-IL-10 (JES5-16E3); and APC-coupled anti-IFN-γ (anti-Interferon-γ, XMG 1.2) and anti-TNF-α (MP6-XT22). As negative controls, PE- or APC-conjugated isotype control Abs were used. To exclude dead cells, Zombie^TM^ UV Fixable Viability Kit (- BioLegend) was used according to the manufacturer’s instructions. The cells were analyzed in an FACS LSR Fortessa (BD Biosciences).

### 3.6. Analysis of Cell Proliferation by Dye Dilution

The proliferation assays were developed using the CellTrace^TM^ Violet Cell Proliferation Kit (Invitrogen-Life Technologies, Carlsbad, CA, USA), for labeling of cells to trace multiple generations using dye dilution by flow cytometry. Fresh cells were stained with CellTrace^TM^ Violet following the manufacturer’s instructions and after the indicated days in culture with the stimuli, the cells were surface stained and analyzed using a BD LSRFortessa flow cytometer (BD Biosciences) with FACSDiva software.

### 3.7. Detection of Spontaneous Apoptosis in Cell Cultures

To assess the effect of nanospheres on the spontaneous apoptosis of DC2.4 dendritic cell line, the cells were cultured in flat-bottom 48-well culture plates containing, or not, sterilized FITC-NanoMBGs at a final concentration of 50 µg/mL. After 24 h of culture at 37 °C and 5% CO_2_, cells were treated with trypsin-EDTA to remove them from the wells and washed. Spontaneous apoptosis was determined by flow cytometry with Annexin V-FITC and propidium iodide using the Annexin V-FITC Kit (according to the manufacturer’s instructions and analyzed in a FACSCanto (BD Biosciences) flow cytometer with FACSDiva software.

### 3.8. Cytokine Quantification

The cytokine content in culture supernatants was assessed using different Ready-SET-Go!® ELISA kits (eBioscience Inc., San Diego, CA, USA) following the manufacturer’s instructions.

### 3.9. Analysis of FITC-NanoMBG-Cell Interaction

Incorporation of FITC-NanoMBGs into spleen cells, BMDCs, DC2.4 cells and Th2 CD4^+^ SR.D10 cell line was analyzed by FACS. Unless otherwise indicated, sterilized NanoMBGs were added to the cell cultures at a final concentration of 50 μg/mL. Where indicated, trypan blue (0.004% final concentration) was added to the cell suspension before analysis, to quench the fluorescence of FITC-nanospheres which could be adsorbed to the outer membrane of the cells. To study the effects of FITC-NanoMBG incorporation on cell size and complexity, forward angle (FSC) and side angle (SSC) scatters were detected by FACS, respectively.

We used different chemical inhibitors to analyze the cell entry mechanisms of the NanoMBGs in dendritic DC2.4 and SR.D10 T cell lines. DC2.4 cells were plated into 24-well plates, grown to confluence during 48 h and then the different inhibitors were added at the indicated concentrations (see below) during 2 h. After that, cells were incubated with the FITC-NanoMBGs for two hours. Then, the cells were treated with trypsin-EDTA to remove them from the wells and washed. A similar procedure was carried out for SR.D10 except that trypsin-EDTA was omitted. Propidium iodide was added to stain dead cells before acquisition of data in a FACSCanto flow cytometer and analysis with FACSDiva software. The inhibitors used were wortmannin (10 µg/mL), cytochalasin B (20 µM), cytochalasin D (4 µM), genistein (3.7 µM) and chlorpromazine (10 µg/mL).

### 3.10. Confocal Microscopy

For confocal microscopy studies, BMDCs were stained 30 min with Hoescht (3 nM, Sigma-Aldrich) and washed twice. Then, the cells were suspended in culture medium and incubated at 37 °C 5% CO_2_ in 8-well glass chambers (Ibidi GmbH, Gräfelfing, Bayern, Germany) for 2 h in the presence, or not, of FITC-NanoMBGs. Stained cells were visualized using a confocal laser scanning microscope TCS SP5II confocal laser scanning microscope (Leica Microsystems, Wetzlar, Germany). FITC fluorescence was excited at 488 nm and measured at 491–586 nm and Hoechst fluorescence was excited at 405 nm and measured at 420–480 nm. Signals from different fluorescent probes were taken in parallel. Immunofluorescence analysis was done using a 63 × 1.4 objective lens. A 0.772 µm thickness section was performed. Image processing was performed using the Fiji ImageJ 1.52p public software.

### 3.11. Statistics

All data are presented as mean ± SEM. Statistical significance was assessed with the two-tailed, unpaired, Student’s t-test. Significance was set at *p*-values *p* < 0.05, *p* < 0.01, *p* < 0.001 and *p* < 0.0001 and it was indicated with asterisks (*, **, *** and **** respectively). All statistical calculations were performed using GraphPad Prism v 8.0 (GraphPad Software, La Jolla California USA, www.graphpad.com) and are referred to the control group or as indicated in the graphs.

## 4. Conclusions

Biomaterials can be potentially sensed by immune cells as “non-self” structures triggering danger signals. This makes it necessary to analyze the interactions of nanoparticles with the immune system for safety issues in clinical applications. Here, the biocompatibility of mesoporous bioactive glass nanospheres in the system SiO_2_-CaO with different types of immune cells has been analyzed. Our study includes key cells connecting innate and adaptive immunity, as dendritic cells; but also primary T and B lymphocytes in charge of cellular and humoral adaptive immune responses. Additional studies using lymphoid and myeloid cell lines ensure a more complete analysis of the interaction of the immune system with these nanospheres. An exhaustive analysis of the specific markers and pro- or anti-inflammatory cytokines of each cell type has been carried out, as well as the molecular mechanisms of entry of these nanoparticles into these different cell types of the immune system. Interestingly, cells from primary and secondary lymphoid organs as well as cultured cells or cell lines can efficiently incorporate NanoMBGs. Incorporation of NanoMBGs does not affect the proliferation or function of the cells analyzed, nor induce inflammatory mediators, pointing to a possible use of this nanomaterial as a safe carrier of active molecules to leukocytes.

## Figures and Tables

**Table 1:** Molecular targets and processes affected by the inhibitors used to analyze the FITC-NanoMBG incorporation into DC2.4 cells.

Name	Effect	References
Wortmannin	Pan-inhibitor of phosphoinositide 3-kinases (PI3-K). It displays a similar potency in vitro for class I, II and III PI3K	[28]
Cytochalasin B and D	Inhibits actin polymerization and network formation by actin filaments. Affect most endocytic pathways.	[29]
Genistein	Tyrosine kinases inhibitor. Inhibits caveolae-mediated endocytosis; also antioxidant, antiangiogenic, and immunosuppressive activities.	[30,31]
Chlorpromazine	Cationic amphipathic drug that blocks clathrin-mediated endocytosis	[32]

## Supplementary Materials

Supplementary Materials can be found at https://www.mdpi.com/1422-0067/21/21/8291/s1.

## Abbreviations

NanoMBGs	Nanospheres of mesoporous SiO_2_-CaO bioactive glass
DC	Dendritic cell
BMDC	Bone marrow derived dendritic cell
APC	Antigen presenting cells
TLR	Toll-like receptors
CC	Click´s culture medium
Ab	Antibody
LPS	Lipopolysaccharide
Poly I:C	Polyinosinic-polycitidylic acid
FCSi	Heat-inactivated Fetal Calf serum
PI3K	Phosphoinositide 3-kinase
PMA	Phorbol 12-myristate 13-acetate
PS-b-PAA	Poly(styrene)-block-poly(acrylic acid)
THF	Tetrahydrofuran
CTAB	Hexadecyl trimethyl ammonium bromide
TEP	Triethyl phosphate
TEOS	Tetraethyl ortosilane
FITC	Fluorescein isothiocyanate
