# Supplementary material for: An Immunological Approach to the Biocompatibility of Mesoporous SiO2-CaO Nanospheres
Source: Int J Mol Sci. 2020 Nov 5;21(21):8291. doi: 10.3390/ijms21218291 (PMC7663838; doi:10.3390/ijms21218291)
Supplement: Supplementary file 1 [file ijms-21-08291-s001.pdf]

## Supplementary data.

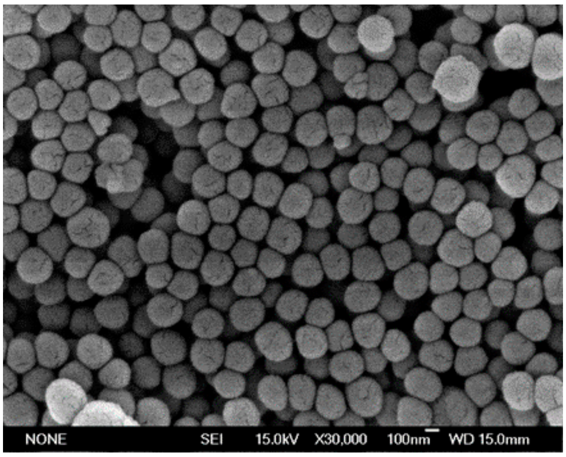


Figure S1. Scanning electron micrograph of NanoMBGs

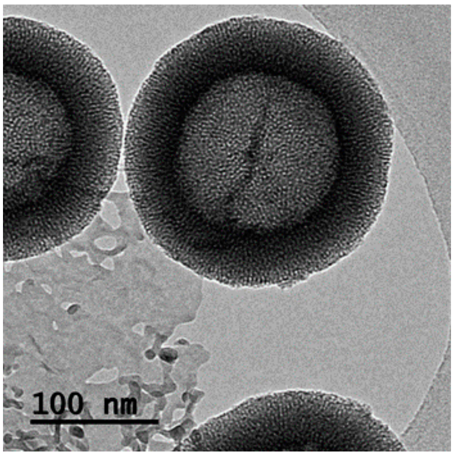


Figure S2. Transmission electron image of NanoMBGs

**Textural parameters obtained by nitrogen adsorption analysis.**

- Single Point Surface Area at P/Po 0.19864939 : **508.7455 $m^2/g$**
- Single Point Adsorption Total Pore Volume of pores less than 72.2143 nm. Diameter at P/Po 0.97264854: **0.435109 $cm^3/g$**

**EDX spectroscopy results.**

The final composition of nanoMBGs was determined as the mean of 15 different measurements

| Spectrum | In stats. | Si | Ca |
|---|---|---|---|
| Nano 1 | Yes | 90.30 | 9.70 |
| Nano 2 | Yes | 82.70 | 17.30 |
| Nano 3 | Yes | 79.88 | 20.12 |
| Nano 4 | Yes | 61.44 | 38.56 |
| Nano 5 | Yes | 76.83 | 23.17 |
| Nano-6 | Yes | 83.14 | 16.86 |
| Nano-7 | Yes | 83.59 | 16.41 |
| Nano 8 | Yes | 87.80 | 12.20 |
| Nano 9 | Yes | 71.53 | 28.47 |
| Nano 10 | Yes | 82.63 | 17.37 |
| Nano 11 | Yes | 82.56 | 17.44 |
| Nano 12 | Yes | 83.87 | 16.13 |
| Nano 13 | Yes | 84.18 | 15.82 |
| Nano 14 | Yes | 85.86 | 14.14 |
| Nano 15 | Yes | 85.36 | 14.64 |
| Mean | | **81.44** | **18.56** |
| Std. deviation | | 7.08 | 7.08 |
| Max. | | 90.30 | 38.56 |
| Min. | | 61.44 | 9.70 |
